# Personality dimensions of patients can change during the course of parkinson’s disease

**DOI:** 10.1371/journal.pone.0245142

**Published:** 2021-01-07

**Authors:** Mathilde Boussac, Christophe Arbus, Julia Dupouy, Estelle Harroch, Vanessa Rousseau, Aurélie Croiset, Fabienne Ory-Magne, Olivier Rascol, Caroline Moreau, Anne-Sophie Rolland, David Maltête, Tiphaine Rouaud, Mylène Meyer, Sophie Drapier, Bruno Giordana, Mathieu Anheim, Elodie Hainque, Béchir Jarraya, Isabelle Benatru, Nicolas Auzou, Lhaouas Belamri, Mélissa Tir, Ana-Raquel Marques, Stephane Thobois, Alexandre Eusebio, Jean Christophe Corvol, David Devos, Christine Brefel-Courbon, on behalf of the PREDI-STIM study group

**Affiliations:** 1 Toulouse NeuroImaging Center, University of Toulouse, Inserm, UPS, Toulouse, France; 2 Psychiatry Department of the University Hospital of Toulouse, CHU Purpan, Toulouse, France; 3 Department of Neurology, Hospital of Avignon, Avignon, France; 4 Department of Clinical Pharmacology and Neurosciences, Parkinson Expert Center, Centre d'Investigation Clinique CIC1436, University Hospital of Toulouse, NeuroToul COEN Center, NS-PARK/FCRIN Network, Toulouse, France; 5 CERPPS—Study and Research Center in Psychopathology and Health Psychology, University of Toulouse II Jean-Jaurès, Toulouse, France; 6 Department of Medical Pharmacology, Neurology and Movement Disorders Department, Referent Center of Parkinson’s disease, CHU of Lille, Univ. Lille Neuroscience & Cognition, Inserm, UMR-S1172, Licend, NS-PARK/FCRIN Network, Lille, France; 7 Department of Neurology, Rouen University Hospital and University of Rouen, Rouen, France; 8 Laboratory of Neuronal and Neuroendocrine Differentiation and Communication, INSERM U1239, NS-PARK/FCRIN Network, Mont-Saint-Aignan, France; 9 Clinique Neurologique, Hôpital Guillaume et René Laennec, NS-PARK/FCRIN Network, Boulevard Jacques Monod, Nantes, France; 10 Neurology Department, Nancy University Hospital, Nancy, France; 11 Behavior and Basal Ganglia Research Unit (EA 4712), University of Rennes 1, Rennes, France; 12 Department of Neurology, Rennes University Hospital, NS-PARK/FCRIN Network, Rennes, France; 13 Service Universitaire de Psychiatrie, Hôpital Pasteur 1, CHU de Nice, Nice, France; 14 Service de Neurologie, Hôpitaux Universitaires de Strasbourg, Strasbourg, France; 15 Institut de Génétique et de Biologie Moléculaire et Cellulaire (IGBMC), INSERM-U964/CNRS-UMR7104/Université de Strasbourg, Illkirch, France; 16 Fédération de Médecine Translationnelle de Strasbourg (FMTS), Université de Strasbourg, NS-PARK/FCRIN Network, Strasbourg, France; 17 Département de Neurologie, Hôpital Pitié-Salpêtrière, AP-HP, Faculté de Médecine de Sorbonne Université, UMR S 1127, Inserm U 1127, and CNRS UMR 7225, and Institut du Cerveau et de la Moëlle épinière, NS-PARK/FCRIN Network, Paris, France; 18 Pôle Neurosciences, Foch Hospital, Suresnes, France; 19 Université de Versailles Paris-Saclay, INSERM U992, CEA Neurospin, France; 20 Service de Neurologie, Centre Expert Parkinson, CIC-INSERM 1402, CHU Poitiers, NS-PARK/FCRIN Network, Poitiers, France; 21 CHU de Bordeaux, Centre Expert Parkinson, Institut des maladies neuro-dégénératives, Bordeaux, France; 22 Hôpital Fondation A de Rothschild, Service de recherche clinique, Paris, France; 23 Department of Neurology, Department of Neurosurgery, Expert Centre for Parkinson's disease, Amiens University Hospital, EA 4559 Laboratoire de Neurosciences Fonctionnelles et Pathologie (LNFP) Université de Picardie Jules Verne, University of Picardy Jules Verne (UPJV), NS-PARK/FCRIN Network, Amiens, France; 24 Neurology Department, Université Clermont Auvergne, EA7280, Clermont-Ferrand University Hospital, NS-PARK/FCRIN Network, Clermont-Ferrand, France; 25 Univ Lyon, Université Claude Bernard Lyon 1, Faculté de Médecine Lyon Sud Charles Mérieux, Lyon, France; 26 CNRS, Institut des Sciences Cognitives, UMR 5229, Bron, France; 27 Centre Expert Parkinson, Hôpital Neurologique "Pierre Wertheimer", Hospices Civils de Lyon, NS-PARK/FCRIN Network, Lyon, France; 28 Aix Marseille Université, AP-HM, Hôpital de La Timone, Service de Neurologie et Pathologie du Mouvement, and UMR CNRS 7289, Institut de Neuroscience de La Timone, NS-PARK/FCRIN Network, Marseille, France; Washington University, St. Louis, UNITED STATES

## Abstract

**Background:**

Studies assessing personality dimensions by the “Temperament and Character Inventory” (TCI) have previously found an association between Parkinson’s disease (PD) and lower Novelty Seeking and higher Harm Avoidance scores. Here, we aimed to describe personality dimensions of PD patients with motor fluctuations and compare them to a normative population and other PD populations.

**Methods:**

All PD patients awaiting Deep Brain Stimulation (DBS) answered the TCI before neurosurgery. Their results were compared to those of historical cohorts (a French normative population, a de novo PD population, and a PD population with motor fluctuations).

**Results:**

Most personality dimensions of our 333 included PD patients with motor fluctuations who are candidates for DBS were different from those of the normative population and some were also different from those of the De Novo PD population, whereas they were similar to those of another population of PD patients with motor fluctuations.

**Conclusions:**

During the course of PD, personality dimensions can change in parallel with the development of motor fluctuations, either due to the evolution of the disease and/or dopaminergic treatments.

## Introduction

Initial studies characterized PD patients as rigid, introverted, obsessional, and depressive. The “Temperament and Character Inventory” (TCI) was subsequently used to examine several PD populations to better assess their personality dimensions. The most recent review of the literature in PD shows that certain specific personality dimensions (lower Novelty Seeking and higher Harm Avoidance scores) differ from those of healthy subjects, based on the TCI and its derivatives (Tridimensional Personality Questionnaire, etc.) [1]. These results reflect the relatively anxious, reflective, and reserved temperament of PD patients. Nevertheless, most studies have been based on small PD samples with a heterogeneous duration of disease (mainly patients in early stages of PD with mild symptoms).

Our main objective was thus to better characterize personality of PD patients. It is why we decided to evaluate personality dimensions in a large cohort of PD patients with motor fluctuations awaiting deep brain stimulation of the sub-thalamic nucleus (DBS-STN) and compare them to those of three historical cohorts (a normative population and two PD populations). This objective was part of a bigger study of which the first part evaluated the association between personality dimensions and quality of life before DBS-STN [2].

## Materials and methods

This is a secondary analysis of our PSYCHO-STIM [2] study, for which the objective was to identify personality dimensions associated with quality of life in PD patients awaiting DBS-STN.

The study population consisted of PD patients who participated in the PREDI-STIM study (https://clinicaltrials.gov/ct2/show/NCT02360683). All patients gave their informed written consent and the PREDI-STIM study was approved by the CPP Nord-Ouest IV Ethical Committee (N° IDRCB: 2013 A0019342).

The TCI assesses the patients’ personality based on seven independent personality dimension scores: four temperament domains (Novelty Seeking (NS), Harm Avoidance (HA), Reward Dependence (RD), and Persistence (P), which is supposed to depend on the cerebral level of dopamine, serotonin, noradrenalin, and glutamate, respectively, according to the original model of C. Robert Cloninger) and three developmental character traits (Self-Directedness (SD), Cooperativeness (C), and Self-Transcendence (ST), which rely on the level of individual, social, and spiritual maturity, respectively).

We selected historical cohorts by first searching for a French normative population in PubMed using the keywords “TCI” and “French population”, to avoid cultural differences in TCI scores, and found only one [3]. We then searched the primary studies with an additional PubMed search using the search terms “Parkinson”, “TCI”, “Novelty Seeking”, “Harm Avoidance”, “Reward Dependence”, “Persistence”, “Self-Directedness”, “Cooperativeness”, and “Self-Transcendence”. After finding only one French PD study [4], we expanded our search to the international level. After removing all meta-analyses or reviews and retaining only studies using the full TCI, nine studies of historical PD cohorts remained. From them, we selected two PD cohorts [4, 5]: a French PD population with motor fluctuations and an early-stage PD population (de novo). The selection criteria of the PD cohorts are presented in “Table 1”. Each historical cohort was selecting only if they had a sufficient number of subjects and if the selected population was well-described.

**Table 1:** Selection criteria of studies on personality in PD population.

Studies	Population	Exclusion criteria	Inclusion criteria
**Kaasinen V,** Nurmi E, Bergman J, et al. Personality traits and brain dopaminergic function in Parkinson’s disease. In: Proceedings of the National Academy of Sciences of the United States of America. Vol 98.; 2001:13272–13277	• never-medicated PD patients (n = 61)	**/**	• never-medicated PD
			• age matched our population (62.1 y.o.)
			• all TCI dimensions scores available
McNamara P, Durso R, Harris E. “Machiavellianism” and frontal dysfunction: evidence from Parkinson’s disease. Cognit Neuropsychiatry. 2007;12(4):285–300	• medicated PD patients (n = 35)	• stage of the PD not known • older than our population (70.4 y.o.)	**/**
Bodi N, Keri S, Nagy H, et al. Reward-learning and the novelty-seeking personality: a between-and within-subjects study of the effects of dopamine agonists on young Parkinson’s patients. Brain. 2009;132(9):2385–2395	• never-medicated PD patients (n = 26) • recently medicated PD patients (n = 22)	• only temperaments scores available (characters scores missing)	**/**
Fassino S, Abbate Daga G, Gramaglia C, et al. Novelty-seeking in Parkinson’s disease after deep brain stimulation of the subthalamic nucleus: a case-control study. Psychosomatics. 2010;51(1):62–67. doi:10.1176/appi.psy.51.1.62	• PD patients treated by DBS-STN (n = 22) • PD patients treated by drugs (n = 22)	• only NS, C and ST dimensions scores available • non-matching DBS-STN surgery criteria and older than our population (68,1 y.o.)	**/**
**Dupouy J.** Personnalité Et Maladie De Parkinson Idiopathique: À Propos D’Une Revue De La Littérature Et De Deux Études Expérimentales. Published online 2014.	• PD patients awaiting DBS-STN (n = 30)	**/**	• PD patients with motor fluctuations awaiting DBS-STN with matching demographic features as our population • all TCI dimensions scores available
Díaz-Santos M, Cao B, Yazdanbakhsh A, Norton DJ, Neargarder S, Cronin-Golomb A. Perceptual, cognitive, and personality rigidity in Parkinson’s disease. Neuropsychologia. 2015;69:183–193. doi:10.1016/j.neuropsychologia.2015.01.044	• medicated PD patients (n = 28)	• TCI dimensions scores non-available • TCI version expanded to 240 items	**/**
Harris E, McNamara P, Durso R. Novelty seeking in patients with right- versus left-onset Parkinson disease. Cogn Behav Neurol. 2015;28(1):11–16. doi:10.1097/WNN.0000000000000047:	• medicated PD patients (2 PD groups: left-onset, n = 17; right-onset, n = 18)	• TCI version expanded to 240 items	**/**
Ishii T, Sawamoto N, Tabu H, et al. Altered striatal circuits underlie characteristic personality traits in Parkinson’s disease. J Neurol. 2016;263(9):1828–1839	• un-medicated and medicated PD patients (n = 16)	• only temperaments scores available (characters scores missing)	**/**
Luca A, Nicoletti A, Mostile G, et al. Temperament traits and executive functions in Parkinson’s disease. Neurosci Lett. 2018;684:25–28. doi:10.1016/j.neulet.2018.06.040	• medicated PD patients (n = 50)	• only NS, HA and RD dimensions scores available • TCI version expanded to 240 items	**/**

PD = Parkinson’s disease; DBS-STN = Deep Brain Stimulation of the Sub-Thalamic Nucleus; y.o. = years old; NS = Novelty Seeking; HA = Harm Avoidance; RD = Reward Dependence; C = Cooperativeness; ST = Self-Transcendence; TCI = Temperament and Character Inventory.

### Statistical analyses

A descriptive analysis was performed on the study population, and missing responses in the TCI, were imputed [2].

First, one-sample Wilcoxon tests were used for each TCI dimension for comparisons of the study population with the two historical cohorts [3, 5], without full data available, using the mean scores of the TCI dimensions of the two cohorts. Two-sample Mann-Whitney tests were used for each TCI dimension for comparison with our previous study [4], for which full data were available. Then, the 95% confidence interval (CI95) was calculated for each population to check for statistical significance: a non-overlap between the CI95 represents a true difference between populations.

Then, multivariate linear regression models were generated to explain the variability of the TCI dimension scores using the TCI dimensions as response variables and three explanatory variables: the LED (levodopa equivalent dose), the presence versus absence of dopaminergic agonists, and the sex of the patient. Seven models were generated (one for each TCI dimension).

Tests were two-sided and the alpha level was set to 0.05. For comparisons with the historical cohorts, only results with a p-value<0.05 and non-overlapping CI95 were considered significant. All analyses were performed using R Studio Software Version 1.1.456.

## Results

Our PD population [2], included 333 PD patients (113 women and 220 men), with a mean age of 61.1±7.2 and a mean duration of PD of 10.2±4.1 years. All patients were under antiparkinsonian treatment at the time of study, with a mean LED of 1.181.6±789.4 mg/day.

From the French normative population [3], we selected data of the “old group” *(256 subjects aged from 50 to 88)* for matching with our PD patients. The PD patients presented significantly higher scores for the NS, RD, P, SD, and C dimensions and significantly lower scores for the ST dimension than the “old group” of normative subjects (“Table 2”). Only the HA was similar between the two populations.

**Table 2:** Comparisons with historical cohorts.

PD patients,			French general population,					De novo PD patients,					PD patients with motor fluctuations,				
PREDI-STIM *(n = 333)*			Pelissolo et al. (2000) [3] *(n = 256)*					Kaasinen et al. (2001) [5] *(n = 61)*					Dupouy (2014) [4] *(n = 30)*				
	Mean	CI	Mean	CI		p.value		Mean	CI		p.value		Mean	CI		p.value	
**NS**	16.8	16.3–17.3	14.5	13.9–15.1	^N-O^	7.4x10^-15^	*	13.1	11.6–14.6	^N-O^	3.0x10^-28^	*	17.8	15.1–20.5		0.46	
**HA**	17.5	16.8–18.2	16.6	15.8–17.4		4.7x10^-2^	*	18.8	17.1–20.5		1.1x10^-3^	*	19.4	16.7–22.2		0.10	
**RD**	15.4	15.1–15.8	14	13.5–14.5	^N-O^	3.6x10^-12^	*	14.1	13.2–15.0	^N-O^	8.1x10^-10^	*	15.5	14.2–16.7		1.00	
**P**	5.4	5.2–5.6	4.6	4.4–4.8	^N-O^	9.8x10^-12^	*	3.51	3.0–4.0	^N-O^	5.8x10^-39^	*	6.2	4.3–8.1		0.98	
**SD**	34.2	33.5–34.9	32.7	32.0–33.4	^N-O^	2.4x10^-7^	*	31.4	29.8–33.0	^N-O^	4.1x10^-17^	*	31.1	28.3–33.9		0.04	*
**C**	33.6	33.2–34.1	32.3	31.7–32.9	^N-O^	1.6x10^-11^	*	32.2	30.9–33.5		1.6x10^-11^	*	33.4	31.8–35.0		0.78	
**ST**	12.2	11.6–12.8	14.6	13.9–15.3	^N-O^	1.0x10^-15^	*	12.7	10.9–14.5		3.4x10^-3^	*	15.2	13.0–17.5	^N-O^	8.9x10^-3^	*

One-sample (for Pelissolo and Kaasinen studies) and two-sample (for Dupouy study) Wilcoxon tests: * p.value < 0.05 –CI = Confidence Interval at a confidence level of 95%–N-O = Non-overlapping CI95%–PD = Parkinson’s disease; NS = Novelty Seeking; HA = Harm Avoidance; RD = Reward Dependence; P = Persistence; SD = Self-Directedness; C = Cooperativeness; ST = Self-Transcendence.

Our PD patients also presented significantly higher scores for the NS, RD, P, and SD dimensions than the de novo PD patients (never-medicated) [5] and significantly lower scores in the ST dimension than the other population of PD patients with motor fluctuations [4] (Table 2).

In the linear models, sex was significantly associated with the HA, RD, C, and ST scores in our PD population (women having higher scores in these dimensions). Quantitative LED and the use of dopaminergic agonists were not associated with any TCI dimensions.

## Discussion

This study shows that PD patients with motor fluctuations awaiting DBS may have a characteristic personality that changes during the course of the disease and the introduction of dopaminergic treatments.

PD patients with motor fluctuations who are candidates for DBS had significantly higher scores for Novelty Seeking, Reward Dependence, Persistence, Self-Directedness, and Cooperativeness and lower scores for Self-Transcendence than the age-matched French normative subjects [3], confirming that this group of PD patients have a specific personality. Nonetheless, our results diverge from the literature which has generally reported lower Novelty Seeking and higher Harm Avoidance scores in PD patients compared to controls [1]. The use of a general population (including subjects with depression, which may mask the higher Harm Avoidance scores of our PD population, as depression is also linked to higher Harm Avoidance scores [6]) as a control group instead of healthy volunteers may explain this divergence in Harm Avoidance scores. Plus, the sex ratio difference between our PD population (women = 33.9%) and the normative population (women = 56.3%) probably explains the higher Self-Transcendence score in the population of Pelissolo, because women generally have higher Self-Transcendence scores than men [3], as confirmed in our population. Finally, the unexpectedly higher Novelty Seeking, Reward Dependence, Persistence, Self-Directedness, and Cooperativeness scores in our PD population appear to be specific to the stage of motor fluctuations and/or dopaminergic treatments, as opposed to the earlier stage of PD populations in the literature [1]. Indeed, De Novo PD patients [5] presented significantly lower personality dimension scores (Novelty Seeking, Reward Dependence, Persistence, and Self-Directedness) than our PD patients with motor fluctuations. The appearance of these differences during the evolution of PD would be unusual, because in Cloninger’s model, temperaments (Novelty Seeking, Reward Dependence, and Persistence) should not change over time in the general population [6], whereas the character (Self-Directedness) may evolve. We propose thus four hypotheses: differences in personality dimensions may i) result from the evolution of PD, with the presence of motor fluctuations, ii) be induced by dopaminergic treatments [7], iii) be linked to decision-making processes concerning surgery, or iv) be related to the stress of awaiting DBS-STN [8].

- During the course of PD, dopaminergic deafferentation extends from the olfactory nucleus to the limbic system [9]. Such brain alterations consequently participate in the emergence of non-motor fluctuations, psychopathologies and impulse-control disorders [10]. Thus, such increasing dopaminergic deafferentation may also affect the personality of PD patients at later stages of the disease. Indeed, the supposed link between TCI temperament dimensions and neurotransmitters [6] appears to be congruent with degeneration of the dopaminergic, serotoninergic, and noradrenergic system in PD [10]. In this supposed link between temperament dimensions and neurotransmitters, Novelty Seeking is associated with dopamine levels in the brain, Harm Avoidance with serotonin, Reward Dependence with noradrenalin, and Persistence with glutamate. These links mainly came from biological and genetic studies [11–18]. Nonetheless, these classical assumptions are not as straightforward. Temperament dimensions seem to be much more complex and to rely on more than a single neurotransmitter. Harm Avoidance scores, for example, were found to be correlated with a dopamine uptake in the right caudate nucleus; whereas the Novelty Seeking scores were not [5]. Finally, it seems that maybe temperament dimensions are not simply linked to neurotransmitters levels but rather to cerebral networks activity. Indeed, Novelty Seeking and Harm Avoidance scores may both be correlated with connectivity between the striatum, hippocampus and amygdale [19]. Striatum connectivity with limbic areas seems therefore to impact personality dimensions [19]. All of this supports the idea that each TCI temperament dimension is a complex concept influenced by different neurotransmitters and/or brain areas, probably explaining why the specific dopaminergic deafferentation of PD impacts several TCI dimensions.
- Dopaminergic treatments may induce changes in personality dimensions, such as an increase in Novelty Seeking scores in PD patients [7], and it seems that only the presence of dopaminergic treatment affects personality dimensions and not the dose nor pharmacological class of the treatment, as seen by the absence of significant association between LED or agonist treatments and personality dimensions. To confirm this result, it would have been interesting to be able to use the complete data from the de novo PD population of Kaasinen and collaborators, which unfortunately was not available. In any case, even if our population was on relatively high dose of LED, it presented a relatively good range of LED (SD = 789.4 mg/day) which should have been enough variability to show an impact of dose of treatment on TCI dimensions. Thus, changes in neurotransmitters levels induced by drugs may modulate some temperament scores [20]. Indeed, many studies have shown that dopaminergic treatments in PD could lead to impulsivity, addiction and risk-taking behaviors, which might be associated with personality dimensions, since personality, behaviors and mood are closed concepts. In fact, PD patients developing pathological gambling generally score higher on novelty-seeking tests [21]. Also, the cerebral pathway implicated in addictive syndromes in PD seems to be mainly the mesocorticolimbic pathway also implied in reward and reinforcement process [22], involved in personality expression. Concerning specifically the dopamine agonists, recently medicated PD patients had higher Novelty Seeking scores compared to controls, whereas never-medicated PD patients had lower Novelty Seeking scores compared to controls [7]. This difference was attributed to a direct effect of dopaminergic agonist [7], even if the reason of these changes was not clearly checked. It could also have appeared with levodopa treatment, as our result does not suggest a class effect of dopaminergic treatment on PD patients personality. Nonetheless, because there is good evidence that dopamine agonists are in part responsible of Impulse Control Disorders development in PD patients [23, 24], a direct causal implication of this class of treatment on personality dimensions cannot be ruled out, even if dopamine agonists are not the only treatment impacting behavioral disorders in PD population. Indeed, Dopamine Dysregulation Syndrome seems to be mainly associated with levodopa uptake [25], and has also been showed to lead to mood changes in PD patients as well as self-injury behaviors [26]. It could thus also probably lead to personality changes. Finally, both levodopa and dopamine agonists could be associated with personality dimensions changes.
- Not all PD patients accept and choose DBS. Thus, PD patients’ personality may influence their choice of a second-generation treatment (DBS versus infusion therapies, for example).
- Certain TCI personality dimensions have been shown to be predictive of a better response to stress (resilience) such as higher Persistence and Self-Directedness [8]. Moreover, resilience is the result of a positive adaptation in the face of adversity, depending on neurobiological mechanisms [27]. It is opposed to vulnerability to stress and depends to the strategy used in response to stress [27]. Thus, the higher scores for the Persistence and Self-Directedness dimensions in our PD population awaiting a stressful event (DBS-STN) relative to those of the de novo PD population may be linked to higher resilience to overcome the stressful event. Moreover, this relation of cause-consequence could be in both ways: either Persistence and Self-Directedness scores increase in order to improve PD patients resilience to deal with the stress of DBS; or only PD patients having enough basal resilience (partly shown by high Persistence and Self-Directedness scores) would choose DBS. In that respect, this second idea would be linked with our preceding hypothesis of DBS choice: maybe, only PD patients that are able to control their stress and demonstrate resilience, are the one accepting DBS.

These observations suggest that the personality dimensions of PD patients may change during the course of the disease. We even make the hypothesis that PD patients personality may not be much different from those of the healthy population at the beginning of the disease. Other factors (pharmacological treatments, evolution of PD, etc.) may be responsible for the observed personality differences. Indeed, in most of the studies that reported differences in the Novelty Seeking and Harm Avoidance scores between PD patients and controls, the PD patients were already being treated with dopaminergic drugs [1]. Even if Harm Avoidance was found increased in a de novo PD population [5], it could only be due to depression, not assessed in this study, and not to the disease itself; which could explain why we did not found any difference in Harm Avoidance scores between our population and the de novo one, depression being present at each stage of the disease.

Comparison of the two PD populations with motor fluctuations awaiting DBS [4], with an equivalent LED, showed similar TCI scores, supporting our four hypotheses. Thus, PD patients with motor fluctuations who are candidates for DBS have a specific personality, the sole small difference in Self-Transcendence likely being related to demographic differences, as Self-Transcendence is the most culturally variable dimension [28].

In conclusion, the personality of PD patients appears to depend on the stage of the disease, with differences due to either the evolution of PD itself, dopaminergic treatments, or psychological factors (the choice of DBS or the stress engendered by it).
